# Association between atherosclerosis and handgrip strength in non‐hypertensive populations in India and Japan

**DOI:** 10.1111/ggi.13312

**Published:** 2018-03-26

**Authors:** Hirotomo Yamanashi, Bharati Kulkarni, Tansy Edwards, Sanjay Kinra, Jun Koyamatsu, Mako Nagayoshi, Yuji Shimizu, Takahiro Maeda, Sharon E Cox

**Affiliations:** ^1^ Department of Island and Community Medicine Nagasaki University Graduate School of Biomedical Sciences Goto Japan; ^2^ Department of Clinical Medicine Institute of Tropical Medicine, Nagasaki University Nagasaki Japan; ^3^ Clinical Division National Institute of Nutrition Hyderabad India; ^4^ Tropical Epidemiology Group London School of Hygiene and Tropical Medicine London UK; ^5^ Department of Non‐communicable Disease Epidemiology London School of Hygiene and Tropical Medicine London UK; ^6^ Department of Community Medicine Nagasaki University Graduate School of Biomedical Sciences Nagasaki Japan; ^7^ School of Tropical Medicine and Global Health Nagasaki University Nagasaki Japan; ^8^ Department of Population Health London School of Hygiene and Tropical Medicine London UK

**Keywords:** atherosclerosis, carotid intima‐media thickness, handgrip strength, pulse wave velocity, sarcopenia

## Abstract

**Aim:**

Although several risk factors contribute to the development of sarcopenia, whether preclinical atherosclerosis contributes to the risk of sarcopenia is not established. The present cross‐sectional study aimed to investigate if there is an association between preclinical atherosclerosis and muscle strength among two ethnic populations.

**Methods:**

Participants included individuals aged ≥40 years and enrolled in the third follow‐up examination of the Andhra Pradesh Children and Parents Study, India, and in the baseline assessments of the Nagasaki Islands Study, Japan. Preclinical atherosclerosis was evaluated by carotid intima‐media thickness, brachial‐ankle pulse wave velocity, cardio‐ankle vascular index. The association of carotid intima‐media thickness and pulse wave velocity/cardio‐ankle vascular index with handgrip strength (HGS) was analyzed separately in the sexes and for hypertensive status from the two cohorts using a multivariable linear regression model.

**Results:**

Data on a total of 1501 participants in India and 3136 participants in Japan were analyzed. Carotid intima‐media thickness was negatively associated with HGS in non‐hypertensive Indian men (B coefficient = −5.38, *P* = 0.036). Arterial stiffness was also associated with HGS in non‐hypertensive Indian men (B = −0.97, *P* = 0.001), but not in hypertensive Indian men. Same as Indian men, we found the significant associations between arterial stiffness and HGS in non‐hypertensive women in both India and Japan (B = −0.44, *P* = 0.020, B = −0.63, *P* = 0.016, respectively), but not in hypertensive women.

**Conclusions:**

The negative association between preclinical atherosclerosis and HGS was dominantly found in non‐hypertensive participants. **Geriatr Gerontol Int 2018; 18: 1071–1078**.

## Introduction

Handgrip strength (HGS) is a simple assessment to estimate overall muscular strength and detect sarcopenia,^1^ and is linked to a range of adverse health outcomes.^2^, ^3^ In a 25‐year prospective study of Japanese American men, low baseline strength at baseline predicted slow gait speed, inability to rise from a chair, self‐care disabilities over the follow‐up period and death.^2^, ^3^ Therefore, investigating the development of sarcopenia and including the use of HGS as a proxy marker might help to avoid the public health burden of associated adverse events.

Previously reported risk factors contributing to the development of sarcopenia include: insulin resistance, mitochondrial dysfunction, physical inactivity, smoking, low 1,25OH vitamin D, low nutritional intake and low protein intake, changes in sex steroid hormones and cytokines associated with aging, and genetic susceptibility.^4^, ^5^, ^6^, ^7^ So far, one Japanese study has reported on preclinical atherosclerosis as a sarcopenia risk factor using carotid intima‐media thickness (CIMT) and brachial‐ankle pulse wave velocity (PWV).^8^ In a cross‐sectional study of 496 Japanese adults, the cross‐sectional area of the thigh muscle corrected by bodyweight measured by computed tomography was negatively associated with PWV in men, but not in women.^8^ In addition, it had no association with CIMT in both men and women. However, these results between measurement tools of preclinical atherosclerosis make it difficult to conclude preclinical atherosclerosis as a risk for developing sarcopenia in the Asian population. Furthermore, we hypothesized that hypertensive status might have an effect on the association between atherosclerosis and HGS, because our previous study implies that hypertensive status inducing vascular endothelial dysfunction might be related to the maintenance of HGS.^9^

Although the aging population in Asian countries is increasing, studies investigated the sarcopenia epidemiology in Asian countries are limited.^10^ Especially in low‐ and middle‐income countries (LMIC), little is known how various cardiometabolic risk factors contribute to sarcopenia progression. Furthermore, a systematic review reported lower HGS in LMIC than in high‐income countries, which implies that the contributing factors for sarcopenia progression might differ between LMIC and high‐income countries.^11^ In our previous birth cohort study in India, HGS and lean body mass at follow up are positively associated with socioeconomic position, energy intake and physical activity level.^12^ However, the relationship between HGS and preclinical atherosclerosis between two ethnic groups has never been investigated.

The present study utilized data from two large cohorts of Asian populations in India and Japan. Simultaneous assessment of these two cohorts enabled us to carry out comparative analyses of a cohort from a high‐income country with that from a LMIC, where confounding structures are likely to differ. The aim of the present study was to investigate the association of preclinical atherosclerosis with HGS, and to assess if this relationship differs in hypertensive status among comparable populations in a LMIC and a high‐income country in Asia.

## Methods

### *Participants*

The participants of the present study consisted of cohort members from two sites. Participants were aged ≥40 years and were included in the third follow up of participants of the Andhra Pradesh Children and Parents Study (APCAPS), or participants were enrolled in the Nagasaki Islands Study (NaIS) during baseline assessments (Figure S1). Details of the selection process and procedures of the examination in APCAPS and NaIS have been published previously.^13^, ^14^ All participants provided written informed consent.

### *Anthropometry*

Height and weight were measured with participants wearing light‐weight clothes and without shoes (Leicester height measure; Chasmors, London, UK, and HD305; Tanita, Tokyo, Japan in APCAPS; BF‐220; Tanita, Tokyo, Japan in NaIS). Body mass index (BMI) was calculated as weight (kg) divided by the square of the height (m). HGS was recorded with the participant in a standing posture, his/her arm extended in a natural position. The handgrip dynamometer was adjusted for the participants so that their second proximal phalanxes were positioned around the handle. The HGS was measured in both hands and the maximum score of all records by both sides was considered for analysis. HGS was measured in the APCAPS group by a Lafayette Hand‐held Dynamometer 78010, (Lafayette Instrument Company, Lafayette, Indiana, USA) (four readings), and in the NaIS group by a Smedley Dynamometer 0‐1019‐01, (Matsumiya Ika Seiki Seisakujo, Tokyo, Japan) (two readings).

### *Preclinical atherosclerosis as measured by CIMT and PWV/cardio‐ankle vascular index*

Measurements of common CIMT by ultrasonography of the right common carotid artery was carried out using an Ethiroli Tiny‐16a (Surabi Biomedical Instrumentation, Coimbatore, India) in APCAPS, and LOGIQ Book XP with a 10‐MHz transducer (GE Healthcare, Milwaukee, WI, USA) in NaIS. The protocol used has been described in detail elsewhere.^13^, ^15^ The mean CIMT was calculated as the mean of right CIMT measurements with carotid plaque excluded. Although AtheroEdge Software in the original APCAPS study and Intima Scope Software (Cross Media, Tokyo, Japan) were used to measure mean CIMT, we re‐evaluated the mean CIMT of APCAPS participants using Intima Scope to avoid the systematic difference of CIMT in the two different types of software.^16^ Arterial stiffness was evaluated as PWV by the Vicorder system (Skidmore Medical Limited, Bristol, UK) in APCAPS, and cardio‐ankle vascular index (CAVI) by the VaSera system (Fukuda Denshi, Tokyo, Japan) in NaIS.

### *Other cardio‐metabolic risk indicators*

Systolic blood pressure and diastolic blood pressure at rest were recorded with a blood pressure measuring device (HEM‐705; Omron, Kyoto, Japan in APCAPS, PASESA AVE‐1500; Shisei Datum, Tokyo, Japan in NaIS). The mean of the three measurements was used in APCAPS. The single measurement was used in NaIS.

We used a questionnaire to obtain information on each participant’s medical history of stroke, ischemic heart disease, medication use for treatment of hypertension, diabetes mellitus, dyslipidemia, smoking status (current smoker, past smoker or never‐smoker) and drinking status (current drinker, no‐drinker).

Blood samples were collected at the time of clinical examination. Serum concentrations of total cholesterol, high‐density lipoprotein cholesterol (HDL‐C), triglycerides (TG), creatinine, fasting plasma glucose, albumin and hemoglobin A1c (HbA1c) were measured by standard laboratory procedures. Total cholesterol was estimated by using the Friedewald–Fredrickson equation in NaIS. The estimated glomerular filtration rate was determined with an established method.^17^

Several variables were defined or calculated from the questionnaire and laboratory results. Hypertension was defined as systolic blood pressure of ≥140 mmHg and/or diastolic blood pressure of ≥90 mmHg or the use of antihypertensive drugs. Diabetes mellitus was defined as an fasting plasma glucose of ≥126 mg/mL, HbA1c of ≥6.5% and/or current use of hypoglycemic drugs. Dyslipidemia was defined as a total cholesterol concentration of ≥220 mg/dL, HDL‐C concentration of <40 mg/dL, TG concentration of ≥150 mg/dL and/or use of lipid‐lowering drugs.

### *Statistical analysis*

Demographic clinical, laboratory and other characteristics were summarized by sex and country. Differences in HGS between populations and sexes were determined using the unpaired *t*‐test or analysis of variance. HGS significantly differed between the populations and by sex, and hence further analyses were stratified by these factors. Hypertension was also investigated as a potential effect modifier through stratified analyses by hypertension status.^9^

To show the relationship of preclinical atherosclerosis as measured by CIMT (or PWV/CAVI) with handgrip, we used multivariable linear regression analysis separately in each country by sex and hypertension. To build the multivariable model, we used a stepwise regression algorithm. All *P*‐values for statistical tests were two‐tailed, with values of <0.05 regarded as statistically significant. Statistical analyses were carried out using stata (version 14.0; StataCorp, College Station, TX, USA).

### *Ethics statement*

The APCAPS study and the NaIS study followed the principles of the Declaration of Helsinki. Ethical approval for the study was obtained in accordance with local institutional requirements (Nagasaki University, Japan, project registration number of 14051404, 141031301; National Institute of Nutrition, India A2/2009).

## Results

### *Cohort profiles*

Table 1 shows a description of the characteristics of participants in each country by sex. Data on a total of 1501 participants in India and 3136 participants in Japan with complete data for variables measured in both country cohorts were analyzed. Compared with the Japanese cohort, the Indian cohort included a higher proportion of men (47% *vs* 39%, *P* < 0.001) and were younger (mean age men 53.5 *vs* 69.9, *P* < 0.001; and women 47.0 *vs* 70.2, *P* < 0.001), had lower HGS (men 25.4 kg *vs* 35.3 kg, age adjusted *P* < 0.001; and women 17.5 kg *vs* 21.1 kg, age adjusted *P* < 0.001), lower CIMT (men 0.66 mm *vs* 0.73 mm, age adjusted *P* < 0.001; and women 0.62 mm *vs* 0.69 mm, age adjusted *P* < 0.001) and a lower proportion of hypertension (men 42.8% *vs* 66.2%, age adjusted *P* < 0.001; and women 28.8% *vs* 62.3%, age adjusted *P* < 0.001).

**Table 1:** Characteristics of participants in each country, by sex

	Indian men	Japanese men	Age‐adjusted *P*	Indian women	Japanese women	Age‐adjusted *P*
No. of participants	703	1,234		798	1,902	
Age, year	53.5 ± 6.6	69.7 ± 10.1		47.0 ± 5.3	70.2 ± 9.4	
Anthropometry						
Height, cm	161.9 ± 6.5	163.2 ± 6.5	<0.001	150.5 ± 5.5	150.4 ± 6.2	<0.001
Weight, kg	54.0 ± 11.4	62.9 ± 9.7	<0.001	49.8 ± 10.4	51.9 ± 8.7	<0.001
Body mass index, kg/m^2^	20.5 ± 3.7	23.6 ± 3.0	<0.001	21.9 ± 4.1	22.9 ± 3.5	<0.001
Handgrip strength, kg	25.4 ± 7.1	35.4 ± 8.2	<0.001	17.5 ± 4.9	21.1 ± 5.3	<0.001
Systolic blood pressure, mmHg	130.0 ± 21.4	136.1 ± 19.2	<0.001	123.3 ± 16.2	137.4 ± 20.1	<0.001
Diastolic blood pressure, mmHg	85.9 ± 15.0	78.6 ± 11.5	<0.001	81.4 ± 11.5	73.6 ± 11.2	<0.001
Hematological parameters						
Fasting plasma glucose, mg/dL	100.1 ± 29.3	NA		97.5 ± 24.9	NA	
Hemoglobin A1c, %	NA	5.76 ± 0.68		NA	5.72 ± 0.50	
Total cholesterol, mg/dL	174.1 ± 38.4	189.8 ± 34.9	<0.001	178.1 ± 37.3	205.8 ± 32.9	<0.001
HDL cholesterol, mg/dL	45.3 ± 15.1	55.4 ± 14.5	<0.001	44.3 ± 12.2	62.1 ± 14.1	<0.001
Triglycerides, mg/dL	150.3 ± 105.9	110.4 ± 77.9	<0.001	129.1 ± 78.3	104.4 ± 57.1	<0.001
Triglycerides‐to‐HDL cholesterol ratio	4.14 ± 5.85	2.26 ± 2.15	<0.001	3.40 ± 3.38	1.87 ± 1.43	<0.001
eGFR, mL/min/1.73 m^2^	69.8 ± 15.2	69.9 ± 15.6	<0.001	71.4 ± 13.7	69.7 ± 14.8	<0.001
Albumin, g/dL	4.7 ± 0.4	NA		4.6 ± 0.4	NA	
Vascular physiology						
Mean carotid intima‐media thickness, mm	0.66 ± 0.14	0.73 ± 0.16	<0.001	0.62 ± 0.11	0.68 ± 0.15	<0.001
Pulse wave verocity‡, m/s	8.04 ± 1.32	NA		7.62 ± 1.23	NA	
Cardio‐ankle vascular index§	NA	8.80 ± 1.29		NA	8.21 ± 1.11	
Specific diseases						
Hypertension	301 (42.8)	811 (65.7)	<0.001	230 (28.8)	1181 (62.1)	<0.001
Anti‐hypertensive drug use	62 (8.8)	569 (46.1)	<0.001	51 (6.4)	829 (43.6)	<0.001
Diabetes mellitus	69 (9.8)	174 (14.1)	0.011	50 (6.3)	170 (8.9)	0.001
Dyslipidemia	404 (57.5)	557 (45.1)	<0.001	420 (52.6)	1114 (58.6)	0.005
History of stroke	10 (1.4)	68 (5.5)	<0.001	9 (1.1)	55 (2.9)	<0.001
History of ischemic heart disease	10 (1.4)	101 (8.2)	<0.001	4 (0.5)	110 (5.8)	<0.001
Life style						
Smoking status			<0.001			<0.001
Current	405 (57.6)	244 (19.8)		3 (0.4)	47 (2.5)	
Former	25 (3.6)	653 (52.9)		0	93 (4.9)	
Never	273 (38.8)	337 (27.3)		795 (99.6)	1762 (92.6)	
Drinking status			<0.001			<0.001
Current drinker	411 (58.5)	728 (59.0)		88 (11.0)	296 (15.6)	
Non	292 (41.5)	506 (41.0)		710 (89.0)	1606 (84.4)	
Nutritional status						
Daily energy intake, kcal	2317.3 ± 839.8	NA		1752.7 ± 596.6	NA	
Daily dietary protein intake, g	49.7 ± 17.3	NA		41.3 ± 14.7	NA	
Physical activity level						
Average energy expenditure, kcal/day	3427.1 ± 652.1	NA		2840.4 ± 412.7	NA	

‡ For data on pulse wave velocity: N = 1465.

§ For data on cardio‐ankle vascular index: N = 2056.

Data are mean ± standard deviation or n (%).

eGFR, estimated glomerular filtration rate; HDL indicates high‐density lipoprotein; NA, not available.

For lifestyle indicators and other cardiometabolic risk indicators, the Indian cohort had lower HDL‐C (men 45.3 mg/dL *vs* 55.4 mg/dL, age adjusted *P* < 0.001; and women 44.3 mg/dL *vs* 62.1 mg/dL, age adjusted *P* < 0.001) and higher TG (men 150.3 mg/dL *vs* 110.4 mg/dL, age adjusted *P* < 0.001; and women 129.1 mg/dL *vs* 104.5 mg/dL, age adjusted *P* < 0.001).

### *Association between CIMT with HGS*

In the multivariable model, CIMT was independently associated with decreased HGS in Indian non‐hypertensive men (B = −5.38, *P* = 0.036; Table 2). As this association was observed only in non‐hypertensive Indian men, we tested effect modification of hypertension toward the slope of CIMT in the multivariable model in the current group. A negative B coefficient in non‐hypertensive Indian men has changed to positive with interaction of hypertensive status (B = −4.96 in non‐hypertensive, 2.66 in hypertensive, *P* = 0.022); the same tendency was observed in Indian women, although it was not statistically significant (B = −0.94 in non‐hypertensive, 3.29 in hypertensive, *P* = 0.203).

**Table 2:** Multivariable linear regression analysis of mean carotid intima‐media thickness and handgrip strength in each country by sex and hypertensive status

		Total		Non‐hypertensive		Hypertensive	
		B coefficient	*P*	B coefficient	*P*	B coefficient	*P*
Indian men	Crude	0.40	0.832	−7.14	0.008	4.76	0.084
	Multivariable model	−0.70	0.687	−5.38	0.036	3.21	0.179
Indian women	Crude	−1.71	0.296	−3.13	0.126	2.16	0.455
	Multivariable model	0.06	0.968	−0.74	0.703	2.85	0.317
Japanese men	Crude	−11.07	<0.001	−16.57	<0.001	−7.61	<0.001
	Multivariable model	−0.41	0.724	−2.39	0.287	0.69	0.613
Japanese women	Crude	−7.67	<0.001	−8.66	<0.001	−6.20	<0.001
	Multivariable model	−0.60	0.399	−1.20	0.390	−0.47	0.566

Covariates included in the multivariable model in Indian men: age, height, body mass index (BMI), high‐density lipoprotein cholesterol, albumin, history of ischemic heart disease, smoking status and use of antihypertensive drugs. In Indian women: age, height, BMI, albumin, history of ischemic heart disease, drinking status and use of antihypertensive drugs. In Japanese men: age, height, BMI, diastolic blood pressure, hemoglobin A1c, total cholesterol, high‐density lipoprotein cholesterol, estimated glomerular filtration rate, history of stroke, drinking status and use of antihypertensive drugs. In Japanese women: age, height, BMI, diastolic blood pressure, high‐density lipoprotein cholesterol, estimated glomerular filtration rate, history of ischemic heart disease and use of antihypertensive drugs.

No association was seen in multivariable analysis in the Japanese cohort.

### *Association between arterial stiffness with HGS*

In the multivariable model in the Indian cohort, an independent negative association between PWV and HGS was observed only in men (B = −0.41, *P* = 0.044), and was not statistically significant in women (B = −0.27, *P* = 0.054; Table 3). Stratified by hypertension, a stronger association in men and significant association in women were found only in non‐hypertensive participants (B = −0.97, *P* = 0.001 and B = −0.44, *P* = 0.020, respectively), whereas no association was observed in hypertensive Indian participants. Effect modification of hypertension was found in the Indian cohort in Indian men (B = −0.89 in non‐hypertensive, 0.03 in hypertensive, *P* = 0.014), although in Indian women it was not significant (B = −0.45 in non‐hypertensive, 0.08 in hypertensive, *P* = 0.069).

**Table 3:** Multivariable linear regression analysis of arterial stiffness and handgrip strength in each country by sex and hypertensive status

		Total		Non‐hypertensive		Hypertensive	
		B coefficient	*P*	B coefficient	*P*	B coefficient	*P*
Indian men	Crude	−0.27	0.180	−0.79	0.012	−0.35	0.232
	Multivariable model	−0.41	0.044	−0.97	0.001	0.12	0.657
Indian women	Crude	−0.22	0.121	−0.43	0.033	0.13	0.562
	Multivariable model	−0.27	0.054	−0.44	0.020	0.08	0.734
Japanese men	Crude	−1.85	<0.001	−2.28	<0.001	−1.55	<0.001
	Multivariable model	−0.26	0.205	0.08	0.855	−0.34	0.158
Japanese women	Crude	−1.20	<0.001	−1.61	<0.001	−0.84	<0.001
	Multivariable model	−0.13	0.361	−0.63	0.016	0.08	0.607

Arterial stiffness was measured by using pulse wave velocity in the Indian cohort, and cardio‐ankle vascular index in the Japanese cohort.

Covariates included in the multivariable model in Indian men: age, height, body mass index (BMI), systolic blood pressure, albumin, history of ischemic heart disease, smoking status, daily energy intake and use of antihypertensive drugs. In Indian women: age, height, BMI, albumin, drinking status and use of antihypertensive drugs. In Japanese men: age, height, BMI, diastolic blood pressure, total cholesterol, HDL cholesterol, estimated glomerular filtration rate, history of stroke, drinking status and use of antihypertensive drugs. In Japanese women: age, height, BMI, diastolic blood pressure, total cholesterol, estimated glomerular filtration rate and use of antihypertensive drugs.

In the multivariable model in the Japanese cohort, no independent association between CAVI and HGS was observed. Stratified by hypertension, however, a negative association was observed in non‐hypertensive Japanese women (B = −0.63, *P* = 0.016), but not in hypertensive women (B = 0.08, *P* = 0.607). In Japanese women, effect modification of hypertension was tested, but not statistically significant (B = −0.19 in non‐hypertensive, −0.10 in hypertensive, *P* = 0.727).

## Discussion

The present study observed a negative association between CIMT and HGS in non‐hypertensive Indian men. Furthermore, arterial stiffness as measured by PWV/CAVI was also negatively associated with HGS in non‐hypertensive Japanese women and non‐hypertensive Indian men and women. Interestingly, these associations were not found in hypertensive participants.

Compared with the Indian cohort, HGS was stronger in the Japanese cohort. Variation in HGS might be explained by nutritional and genetic factors.^11^ Exploring differences between the cohorts with different progression of HGS weakness could contribute to a better understanding of the determinants of successful healthy aging. Despite the younger cohort, Indian participants had lower HGS, lower HDL‐C and a higher TG‐to‐HDL‐C ratio, which is a known risk factor for preclinical atherosclerosis in our previous report,^18^ and might indicate insulin resistance,^19^ a known risk factor for sarcopenia.^4^, ^20^ Thus, a higher TG‐to‐HDL‐C ratio could partly explain the difference in HGS between the two cohorts.

One of the interesting findings of the present study, that most of associations were found in limited to non‐hypertensive participants, should be addressed. A prospective study of 195 community‐dwelling Dutch older men reported higher baseline CIMT to be associated with lower HGS after 4‐year follow up.^21^ That study took into account the confounding factors of age, BMI, fat mass, smoking status, use of antihypertensive drugs, systolic blood pressure, diastolic blood pressure and educational level. However, similar to the present study, cross‐sectional analysis of CIMT and HGS did not find a statistically significant association using baseline data. Another cross‐sectional study of 400 Dutch men aged 40–80 years reported that CIMT and PWV were not associated with HGS after adjustment for relevant confounders, such as age, educational level, comorbidity, use of antihypertensive drugs, smoking and use of alcohol.^22^ Even though both of the above studies included antihypertensive drugs as a confounder, it is possible that opposite associations in the hypertensive and non‐hypertensive sub‐populations might underlie the apparent lack of association between CIMT and PWV in these studies. In the current study, hypertension had a significant interaction between CIMT/PWV in Indian men. Furthermore, hypertension is a strong factor in the development of preclinical atherosclerosis and also induced endothelial dysfunction, which might relate to the background mechanism and explain our contrasting results by hypertensive status.^23^

Several possible explanations for an association between atherosclerosis and muscle strength are possible. First, inflammation could be an underlying mechanism for atherosclerosis, as well as sarcopenia. Several studies have reported inflammatory cytokines and markers (interleukin‐6, tumor necrosis factor alpha, C‐reactive protein) to be inversely associated with muscle mass and strength.^5^, ^6^ Inflammatory cytokines could alter blood vessel dynamics, which in turn can alter muscle metabolism and skeletal muscle breakdown.^24^ Second, insulin resistance might be a common pathway for both atherosclerosis and muscle loss. Skeletal muscle is the main site for insulin‐mediated glucose disposal. High insulin resistance is linked with atherosclerosis.^25^ In addition, insulin resistance is inversely associated with muscle mass.^20^

However, these explanations cannot explain why we only observed the inverse association between preclinical atherosclerosis and HGS in non‐hypertensive but not hypertensive participants. Hypertension is a well known risk factor for endothelial impairment.^26^ Although endothelial impairment, as evidenced by reduced flow‐mediated dilation, has been associated with skeletal muscle mass measured by dual‐energy X‐ray absorptiometry in Brazilian older people.^27^ Participants with the lowest tertile of flow‐mediated dilation had a higher risk of sarcopenia defined by skeletal muscle mass index (odds ratio 5.44, 95% CI 1.22–24.24).^27^ Hence hypertension‐induced endothelial dysfunction might exacerbate muscle wasting. Therefore, in non‐hypertensive participants, age‐related endothelial dysfunction might induce atherosclerosis and, in turn, exacerbate muscle wasting.

In the hypertensive population, hypertensive‐induced endothelial injury might not always induce atherosclerosis, because age‐related bone marrow activities, which regulate CD‐34 positive cells and activated platelet cells, are not the same.^28^ We have previously shown that circulating CD34‐positive cells, an indicator of vascular repair in hypertension‐induced endothelial dysfunction, are associated with increased HGS in hypertensive older Japanese men.^9^, ^23^ Furthermore, a positive association between HGS and preclinical atherosclerosis using CIMT was observed only in participants with higher platelet counts, an indicator of vascular repair activity.^23^, ^28^ Then, endothelial injury might induce muscle wasting if activities of CD34‐positive cells and platelet cells are insufficient. In contrast, endothelial injury could induce atherosclerosis and also angiogenesis (increased collateral blood flow), which leads to maintenance of HGS if the activities are aggressive. Therefore, in hypertensive participants, the association between HGS and atherosclerosis might be canceled out between the individuals with aggressive and insufficient bone marrow activities.

Inconsistent results of the associations between preclinical atherosclerosis and muscle strength among two ethnic cohorts also should be addressed. Abdominal obesity might confound the association between preclinical atherosclerosis and muscle strength. Abdominal obesity, measured by waist‐to‐height ratio, was associated with CIMT in a Chinese cross‐sectional study.^29^ High waist circumference was associated with lower HGS in a British cross‐sectional study.^30^ The present Japanese cohort also had higher bodyweight and waist circumference compared with the Indian cohort, and a higher proportion of abdominal obesity is expected, which could modify the relationship.

The present study had several strengths. First, we had a large sample size from two different population‐based cohorts to analyze the associations. Second, our study had detailed measurements of potential confounders including anthropometry, lifestyle indicators and other cardiometabolic risk indicators. Finally, the study participants were of a broad age range. Some limitations of the present study should be mentioned. As this was a cross‐sectional study, we were not able to establish cause–effect relationships. Differences in methods for measures of exposure, outcome and potential confounders might influence the results.

Hypertension appears to modify the association between preclinical atherosclerosis and HGS. Further study to elucidate the mechanism for the association between atherosclerosis and muscle maintenance depending on hypertensive status could help to develop preventive strategies for HGS weakness.

## Disclosure statement

The study was funded by a Grant‐in‐Aid for Scientific Research from the Japan Society for the Promotion of Science (No. 22370090), Kanae Foundation for the Promotion of Medical Science, and Meiji Yasuda Life Foundation of Health and Welfare. The third survey wave of Andhra Pradesh Children and Parents Study was funded by the Wellcome Trust (No. 084774) with infrastructural support from the National Institute of Nutrition (Indian Council for Medical Research). The financial sponsor played no role in the design, execution, analysis and interpretation of data, or writing of the study.

HY, BK, SK, SE report grants from Kanae Foundation for the Promotion of Medical Science, and Meiji Yasuda Life Foundation of Health and Welfare. TM reports grants from a Grant‐in‐Aid for Scientific Research from the Japan Society for the Promotion of Science (No. 22370090). The other authors declare no conflict of interest.

[Correction added on 6 June 2018, after first online publication: The Disclosure statement has been updated for the complete list of funders.]

## Supporting information


**Figure S1** Flow chart for participation in the study.
