# Supplementary figures and images for: Association between atherosclerosis and handgrip strength in non‐hypertensive populations in India and Japan
Source: Geriatr Gerontol Int. 2018 Mar 26;18(7):1071–8. doi: 10.1111/ggi.13312 (PMC6144064; doi:10.1111/ggi.13312)

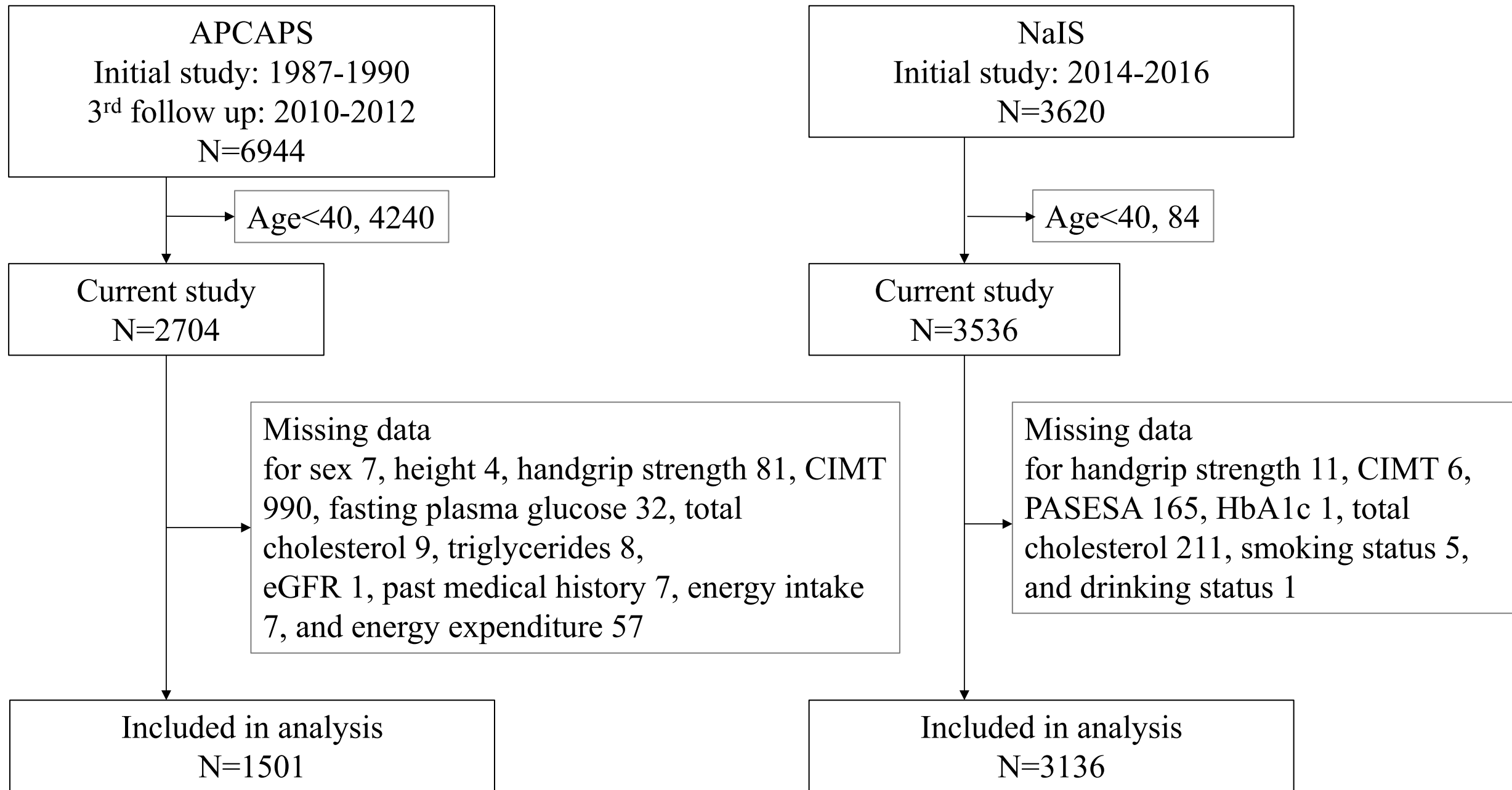

**Figure 1 Flow chart for participation in the study.**

Supplement: Supplementary file 1 — Figure S1 Flow chart for participation in the study. [file GGI-18-1071-s001.pdf]
